# Low level of polyandry constrains phenotypic plasticity of male body size in mites

**DOI:** 10.1371/journal.pone.0188924

**Published:** 2017-11-30

**Authors:** Peter Schausberger, Andreas Walzer, Yasumasa Murata, Masahiro Osakabe

**Affiliations:** 1 Department of Behavioural Biology, University of Vienna, Vienna, Austria; 2 Group of Arthropod Ecology and Behavior, Department of Crop Sciences, Vienna, Austria; 3 Laboratory of Ecological Information, Graduate School of Agriculture, Kyoto University, Sakyo-ku, Kyoto, Japan; Université de Sherbrooke, CANADA

## Abstract

Polyandry, i.e. females mating with multiple males, is more common than previously anticipated and potentially provides both direct and indirect fitness benefits to females. The level of polyandry (defined by the lifetime number of male mates of a female) is an important determinant of the occurrence and intensity of sexual selection acting on male phenotypes. While the forces of sexual selection acting on phenotypic male traits such as body size are relatively well understood, sexual selection acting on phenotypic plasticity of these traits is unexplored. We tackled this issue by scrutinizing the link between polyandry and phenotypic plasticity of male body size in two sympatric plant-inhabiting predatory mite species, *Phytoseiulus persimilis* and *Neoseiulus californicus*. These two species are similar in life history, ecological niche requirements, mating behavior, polygyny and female body size plasticity but strikingly differ in the level of both polyandry and phenotypic plasticity of male body size (both lower in *P*. *persimilis*). We hypothesized that deviations from standard body size, i.e. the size achieved under favorable conditions, incur higher costs for males in the less polyandrous *P*. *persimilis*. To test our hypotheses, we conducted two experiments on (i) the effects of male body size on spermatophore transfer in singly mating females and (ii) the effects of mate sequence (switching the order of standard-sized and small males) on mating behavior and paternity success in doubly mating females. In *P*. *persimilis* but not *N*. *californicus*, small males transferred fewer but larger spermatophores to the females; in both species, females re-mated more likely with standard-sized following small than small following standard-sized males; in *P*. *persimilis*, first standard-sized males sired a higher proportion of offspring produced after re-mating by the female than first small males, whereas in *N*. *californicus* the paternity success of small and standard-sized males was independent of the mating sequence. Based on our results and pertinent previous studies, which showed that females of *P*. *persimilis*, but not *N*. *californicus*, prefer mating with standard-sized over small males and allow them fertilizing more eggs, the lack of interspecific difference in female body size plasticity, and the absence of any clue pointing at a role of natural selection, we suggest that the interspecific difference in male body size plasticity is sexually selected. Our study provides an indication of sexual selection constraining plasticity of male phenotypes, suggesting that the level of polyandry may be an important co-determinant of the level of phenotypic plasticity of male body size.

## Introduction

Polyandry, females mating with multiple males, is more widespread than previously anticipated because of its potential to provide not only direct but also indirect fitness benefits to females [1–3]. The level of polyandry varies within and between species and determines the opportunities and intensity of sexual selection acting on male phenotypic traits before and after mating. Multiple mating by females promotes post-copulatory sexual selection via cryptic female choice [4] and sperm competition [5], whereas monandry and rare re-mating by females promote pre-copulatory sexual selection via male-male competition and female choice [6,7]. Across animals, observed levels of polyandry, defined by the lifetime number of male mates, ranges up to a maximum of more than 100 male mates [8]. The level of polyandry affects, for example, the operational sex ratio (OSR), that is, the ratio between sexually active, ready-to-mate males and receptive females [9–11]. Everything else being equal, the lower the level of polyandry, the more male-biased the OSR, the fewer the opportunities for individual males to obtain a mate. Accordingly, sexual selection acts more strongly on male phenotypic traits in male-biased than female-biased OSRs and in species with low than high female mating rates, which is empirically well-evidenced for both vertebrates [12,13] and invertebrates [11,14]. Here, we scrutinize the link between polyandry and phenotypic plasticity of a key male trait, which is subject to both natural and sexual selection, that is, body size.

Male body size is a prime phenotypic trait in both pre- and post-copulatory sexual selection [15–17]. Before copulation, in many animals, large males are favored over small males in both intrasexual and epigamic selection [18–20] due to superior male-male competitive abilities coupled with female preference for large size [21,22]. Post copulation, larger males are often better in mate guarding, for example by preventing access of rival males to females willing to re-mate, or have advantages in sperm competition or cryptic female choice (i.e., females internally manipulating relative male fertilization success), because of transferring more sperm or nutrients accompanying sperm [23]. While the forces of sexual selection shaping male body size are relatively well understood. with numerous studies documenting the benefits, costs and limits of body size plasticity shaped by natural selection [24–27], the forces of sexual selection on phenotypic plasticity of male body size have been barely explored [28].

Phenotypic plasticity, i.e., phenotypic variation of the same genotype in response to variable environments, is heritable and thus subject to evolution [26]. Being able to adjust the phenotype to local conditions is mostly adaptive, especially in fluctuating or heterogenous environments, by allowing to produce an advantageous phenotype-environment match [24,29]. Environmentally induced (diet, abiotic conditions, predation risk, etc.) variation of phenotypic traits that are also important, or directly involved, in sexual selection such as body size, pheromones, or pigments is common [30–32], but whether sexual selection promotes or constrains the plasticity of these traits is poorly known. Natural selection for high plasticity of sexually relevant traits should be constrained by the costs of high plasticity in sexual selection [33]. Selection will act on plasticity reduction if male phenotypes deviating from standard/optimum have disproportionally low mating and paternity success, and this disadvantage in sexual selection outweighs the potential benefits of higher plasticity favored by natural selection. For example, regarding body size, a well-developed ability to adjust body size to the prevailing food conditions (high plasticity) would be (natural) selectively advantageous in environments with fluctuating food availability in the long term but predictably matching food conditions in the short term, i.e. during the juvenile and adult life phases. However, if females use male body size as an indicator of mate quality, sexual selection would oppose natural selection and constrain the level of body size plasticity.

We addressed the interplay between polyandry and phenotypic plasticity of male body size in two species of sympatric plant-inhabiting predatory mites, *Phytoseiulus persimilis* and *Neoseiulus californicus*. Both species reproduce sexually, have, at large, similar mating behaviors (with competing males actively searching for females but, after mate encounter, females having control whether mating takes place or not [34]), are pseudo-arrhenotokous (only daughters are diploid and carry both the maternal and paternal chromosome set; sons become haploid after losing the paternal chromosome set [35]), and have highly polygynous males with single males fertilizing up to 40 females and siring up to 1500 offspring [36]. Strikingly, the two species differ in both the level of polyandry and the level of phenotypic plasticity of male body size [36–38]. *P*. *persimilis* females have low mating rates, with two mates per female at maximum [39,40], whereas *N*. *californicus* females have moderate mating rates, with up to seven mates per female [41]. As a consequence of differing polyandry levels and at similar tertiary sex ratios (65–80% females) [42], OSR is more strongly biased towards males in *P*. *persimilis* than *N*. *californicus*. Body size is an important trait in sexual selection of both species, both are sexually dimorphic with large females and small males. Female body size is similarly plastic in both species but male body size is less plastic in *P*. *persimilis* than *N*. *californicus* [34,36,37]. At similar mate encounter rates, lower female mating rates should correlate with higher female choosiness and intensify selection of sexually relevant male phenotypic traits and their plasticity [1–3,6,7]; accordingly, in binary choice situations, singly mating *P*. *persimilis* females prefer larger-sized males whereas singly mating *N*. *californicus* females are indiscriminate [34]. Multiple mating by females may provide opportunities to offset or reverse male size disadvantages, likely accruing if females mate only once, but this has not yet been tested for *P*. *persimilis* and *N*. *californicus*.

Broadly defined, we hypothesized that sexual selection acts more strongly on male body size plasticity in *P*. *persimilis* than *N*. *californicus*. The (natural) selectively advantageous level of plasticity of male body size should be constrained by the costs of deviation from standard body size in sexual selection. These costs should be higher in the less polyandrous *P*. *persimilis*, by providing fewer opportunities for males to compensate for disadvantages in single matings, than in the moderately polyandrous *N*. *californicus*. We tested our hypotheses by quantifying the costs of deviation from standard body size in terms of spermatophore transfer, and mating and paternity success (i.e., the relative success in fertilizing eggs) of male mates of polyandrous females sequentially offered first small and second standard-sized or first standard-sized and second small males.

## Materials and methods

### Species origin, isofemale lines and paternity analyses

Experimental animals derived from laboratory-reared populations of *P*. *persimilis* and *N*. *californicus*, founded with specimens collected in the region of Trapani, Sicily [37]. The stock populations of the two species were reared on separate acrylic tile arenas (15 x 15 cm) resting on water-saturated foam cubes in plastic boxes (20 x 20 x 6 cm) half-filled with water. Two-spotted spider mites *T*. *urticae* were provided as prey three times per week by piling up spider mite-infested bean leaves on arenas (for details see [38]). In experiment 1, experimental animals were offspring of females randomly withdrawn from the stock populations. In experiment 2, experimental animals were offspring of females withdrawn from isofemale lines created from the stock populations (establishment and characteristics of isofemale lines described in [38]). Use of isofemale lines that were equipped with unique sets of alleles, and were thus clearly discernible from other lines of the same species, allowed offspring genotyping, and paternity analysis of first and second male mates, in doubly mating females. For paternity analysis, we followed exactly the same protocol as described by [38]. In brief, DNA samples of female offspring of doubly mated females were prepared as crude extracts and directly applied to PCR amplifications (TaKaRa PCR Thermal Cycler TP600; Takara Bio Inc.) following fragment analyses (ABI 3130–200 genetic analyzer with GeneMapper version 3.5; Applied Biosystems). Subsequently, we genotyped the polymorphic microsatellite loci [PP003 (accession no.: LC017803) and PP005 (LC17804) for *P*. *persimilis*; NC019 (LC017805) and NC030 (LC017806) for *N*. *californicus*] and determined paternity of each offspring by comparing the genotypes with their parents.

### (Pre-)experimental units

Cavities laser-cut into rectangular acrylic plates (8 x 3.5 x 0.3 cm) were used for generating small and standard-sized males and for the behavioral assays in both experiments. Each cavity consisted of a cylindrical cell of 1.5 cm Ø and 0.3 cm height closed at the bottom by a gauze and on the upper side by a microscope slide [43]. For oviposition by females whose eggs were used in the experiments, and rearing offspring to adulthood in experiment 2, we used detached spider mite-infested bean leaves placed upside down on water-saturated foam cuboids (6 x 6 x 4 cm) in plastic boxes (10 x 10 x 6 cm) half-filled with water [38].

### Generating virgin females, and small and standard-sized males

Ten females each of *N*. *californicus* and *P*. *persimilis* were randomly withdrawn from the rearing units (experiment 1) or isofemale lines (experiment 2) and placed on separate spider-mite infested bean leaf arenas to obtain predator eggs for generating virgin females and small and standard-sized males. The eggs were collected after 24 h, placed singly in acrylic cages and provided with either limited (10 for *P*. *persimilis*, 8 for *N*. *californicus*) or ample (40 for either predator) spider mite eggs as prey. Limited prey supply differed between the two predators because of species-specific prey demands; 10 and 8 spider mite eggs per capita represent the minimum prey thresholds allowing optimal survival and development to adulthood [37]. The developmental progress of the predators was checked every 24 h until they reached maturity. Sex-specific body size differences—females are about one third larger than males—were used to determine the sex of adult individuals. Virgin females reared under ample prey and virgin males reared under limited and ample prey supply were used as experimental individuals in both experiments. According to their rearing history, males reared under limited prey were considered small males and those reared under ample prey were considered standard-sized males [37]. Within species, standard-sized and small males, reared under ample and limited prey supply, do not differ in survival, longevity and potential number of virgin females fertilized under surplus female availability [36].

### Male mate body size effects on mating duration, spermatophore number and size, and ejaculate mass in singly mating females (experiment 1)

A single virgin female of *P*. *persimilis* or *N*. *californicus* was placed in an acrylic cage, which had been previously loaded with surplus spider mite eggs using a fine marten’s hair brush, together with a conspecific small or standard-sized virgin male. The mating behavior of the couple was observed every 15 min until mating ceased. Males and females were considered mating (copulation *sensu stricto*) when the male was underneath the female in the venter-to-venter position [34,36]. The experiment was replicated 10 to 14 times per type of couple (small or standard-sized male) per species (*P*. *persimilis* and *N*. *californicus*). Following mating, each male and female was mounted in a drop of Hoyer’s medium on a microscope slide [44] to determine the dorsal shield length of the mates, an indicator of their body size [45], and the number and size (diameter) of the transferred spherical spermatophores [39]. The ejaculate mass transferred per mating event was determined by calculating the volumes of the transferred spermatophores ($V\;=\;\frac{4}{3}\pi r^{3}$) and multiplying them by the number of spermatophores.

### Male mate body size and sequence effects on mating behavior, egg production and paternity in doubly mating females (experiment 2)

To consider the species-specific re-mating periods [38], single virgin females of *P*. *persimilis* and *N*. *californicus* were offered single small or standard-sized male mates during two time periods: on days 1 to 4 and on days 11 to 14 after reaching adulthood. The couples were placed in acrylic cages (day 1) and their behavior was observed every 10 min within 6 h to record the occurrence of mating, the mating latency, i.e. the time elapsed between offering a male to the female and beginning of copulation, and the mating duration. If the first mating did not take place within 6 h, the male was removed and another virgin male was added the next day (day 2). Every female mated on day 1 or 2; every male mate was removed immediately after finishing copulation, i.e., when they had separated from females. One day after the first mating (day 2 or 3) a second virgin male, belonging to the other size class than the first male, was offered to the female. This procedure was repeated one day later (day 3 or 4), if no second mating occurred. Only for those females that had not yet mated two times within days 1 to 4, another single virgin male was offered 10 days after the first mating (day 11 or 12). If no mating occurred, another male was offered one day later (day 13 or 14). First and second male mates of a given female derived from different isofemale lines. All mated females were singly placed on an oviposition arena and allowed to lay eggs until oviposition ceased naturally (three consecutive days without egg production). Eggs were daily counted and transferred to the offspring rearing units. After reaching adulthood, the offspring were sexed and daughters embedded in 95% alcohol in Eppendorf tubes to be later subjected to paternity analysis. The experiment was replicated 27 to 41 times per mating sequence (first small, second standard-sized and first standard-sized, second small) and species (27 and 39 replicates for *P*. *persimilis*; 30 and 41 replicates for *N*. *californicus*). Daughters of 22 and 17 doubly mated females of *P*. *persimilis* (4 to 52 daughters per female) and *N*. *californicus* (4 to 29 daughters per female) could be successfully genotyped.

### Statistical analyses

SPSS 23 (IBM Corp., 2015) was used for all statistical analyses. In experiment 1, we used generalized linear models (GLM) to analyze the effects of prey supply during juvenile development (limited, ample) and species (*P*. *persimilis*, *N*. *californicus*) on adult male body size. Least significant difference (LSD) tests were used for post-hoc pairwise comparisons. For each species separately, we used bivariate linear correlations to analyze the relationship between male dorsal shield length and mating duration, spermatophore size, and total ejaculate mass, and Spearman rank correlation for the relationship between male dorsal shield length and spermatophore number. In experiment 2, we used separate GLMs to evaluate the influence of species (*P*. *persimilis*, *N*. *californicus*) and mating sequence of small and standard-sized males on the day (Poisson, log link), latency (Gamma, identity link) and duration (normal, identity link) of the first and second mating and the total number of eggs produced (normal, identity link) by doubly mated females. LSD tests were used for post-hoc pairwise comparisons if needed. Binary logistic regression was used to assess the influence of species and mating sequence on the re-mating likelihood (yes/no). The influence of species and mating sequence of small and standard-sized males on offspring production (normal, identity link; here using the data of all doubly mated females) and the proportion of daughters sired by first mates after re-mating (binomial, counts of events; here only using the data of doubly mated females whose daughters were subjected to paternity analysis) were analyzed by separate GLMs. LSD and Sidak tests were used for post-hoc pairwise comparisons.

## Results

### Phenotypic plasticity of male body size

Males of both species (GLM: *Wald ӽ*_*1*_^*2*^ = 3.518, *P* = 0.06) reared under limited prey were smaller than males reared under ample prey (*Wald ӽ*_*1*_^*2*^ = 147.239, *P* < 0.001). The significant interaction between species and prey supply (*Wald ӽ*_*1*_^*2*^ = 5.139, *P* = 0.02) indicates that *P*. *persimilis* (μm, mean ± SE; 277.50 ± 1.27) and *N*. *californicus* males (277.93 ± 1.27) were similarly sized when provided with ample prey during juvenile development (LSD; *P* = 0.78); in contrast, males of *N*. *californicus* (259.07 ± 1.45) grew smaller than those of *P*. *persimilis* (264.50 ± 1.23) when provided with limited prey (LSD; *P* = 0.004), confirming lower male body size plasticity in *P*. *persimilis* than *N*. *californicus*.

### Male mate body size effects on mating duration, spermatophore number and size, and ejaculate mass in singly mating females (experiment 1)

**Fig 1:**
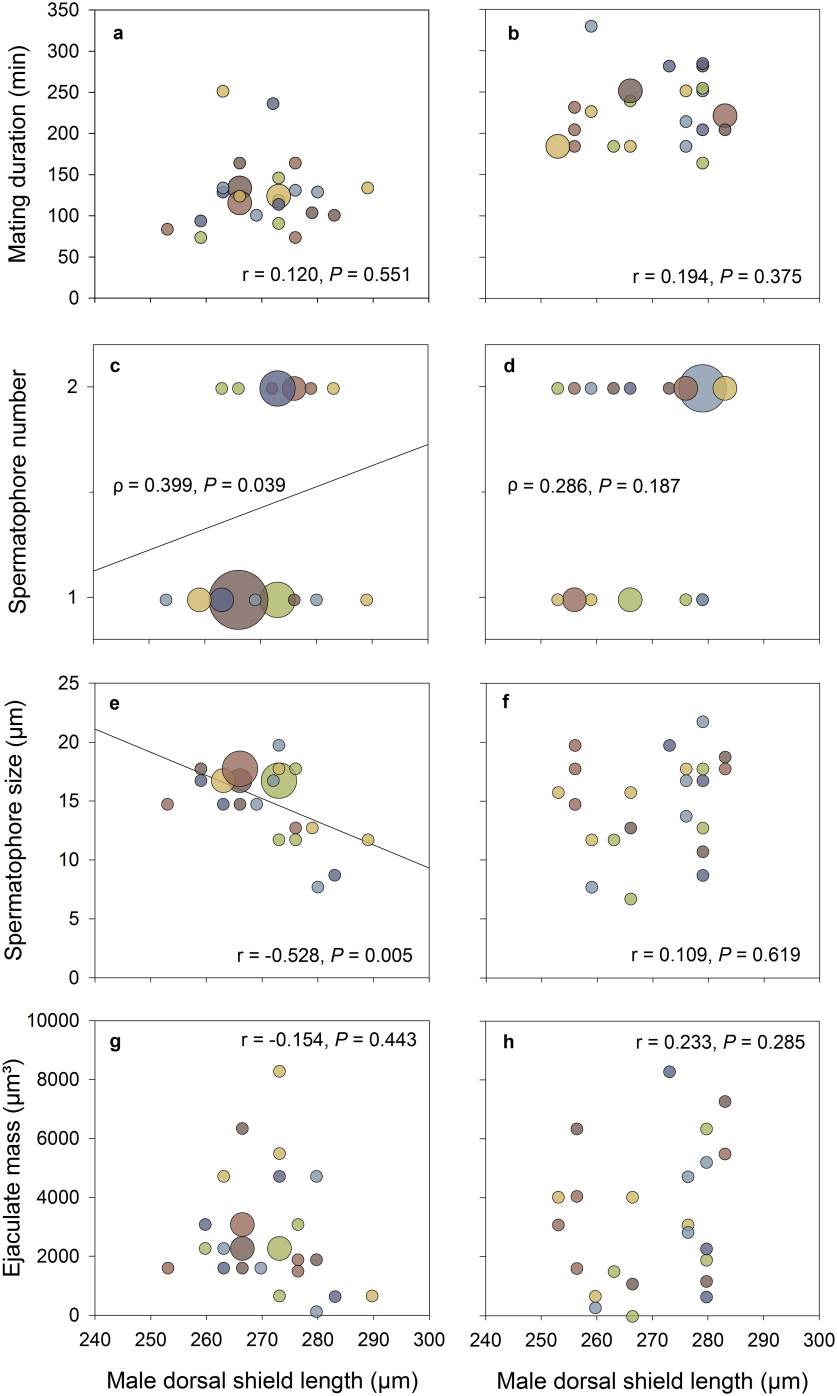
Correlations between male body size and mating duration, spermatophore number and size, and ejaculate mass. Mating duration (a, b), spermatophore number (c, d), spermatophore size (e, f) and ejaculate mass (g, h) regressed on male body size of *P*. *persimilis* (a, c, e, g; *n* = 27) and *N*. *californicus* (b, d, f, h; *n* = 23). Symbol size is proportional to sample size. Statistical results inside panels refer to bivariate linear (a, b, e, f, g, h) and Spearman rank (c, d) correlations.

In *P*. *persimilis*, male body size did not affect mating duration (Fig 1a) but was positively correlated with the number of spermatophores transferred per mating event (Fig 1c) and negatively correlated with spermatophore size (Fig 1e). Small males compensated for lower spermatophore number by producing larger spermatophores, resulting in similar ejaculate mass transferred per mating event by small and standard-sized males (Fig 1g). In *N*. *californicus*, male body size did not affect mating duration, spermatophore number, spermatophore size, and ejaculate mass (Fig 1b, 1d, 1f and 1h).

### Male mate body size and sequence effects on mating behavior, egg production and paternity success in doubly mating females (experiment 2)

The re-mating likelihood did not differ between *P*. *persimilis* and *N*. *californicus* females sequentially offered differently sized males (binary logistic regression: *Wald ӽ*_*1*_^*2*^ = 0.007, *P* = 0.93) but the females of both species re-mated more likely with a standard-sized following a small male (proportion re-mating: *P*. *persimilis* = 1.00, *N*. *californicus* = 0.96) than a small following a standard-sized male (proportion re-mating: *P*. *persimilis* = 0.71, *N*. *californicus* = 0.75) (*Wald ӽ*_*1*_^*2*^ = 7.906, *P* = 0.005).

**Fig 2:**
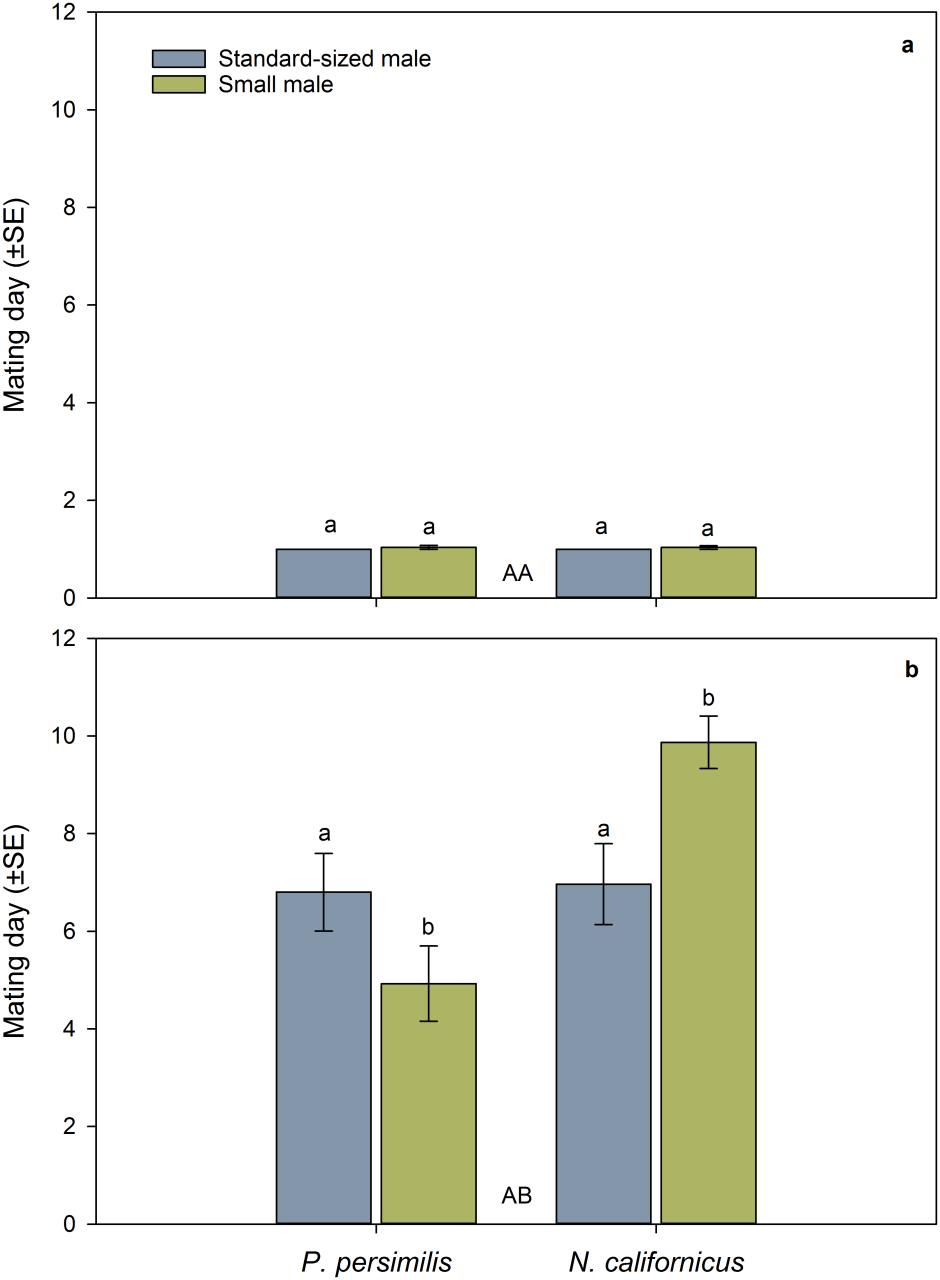
Influence of male body size and mating sequence on mating dates. First (a) and second (b) mating date of doubly mating *P*. *persimilis* (*n* = 52) and *N*. *californicus* (*n* = 49) females, according to the mating sequence of small and standard-sized males. Different capital letters between bars refer to differences between species across mating sequences (GLM; *P* < 0.001). Different lower case letters on top of bars indicate significant differences between small and standard-sized male mates within species (Sidak following GLM; *P* < 0.05).

**Fig 3:**
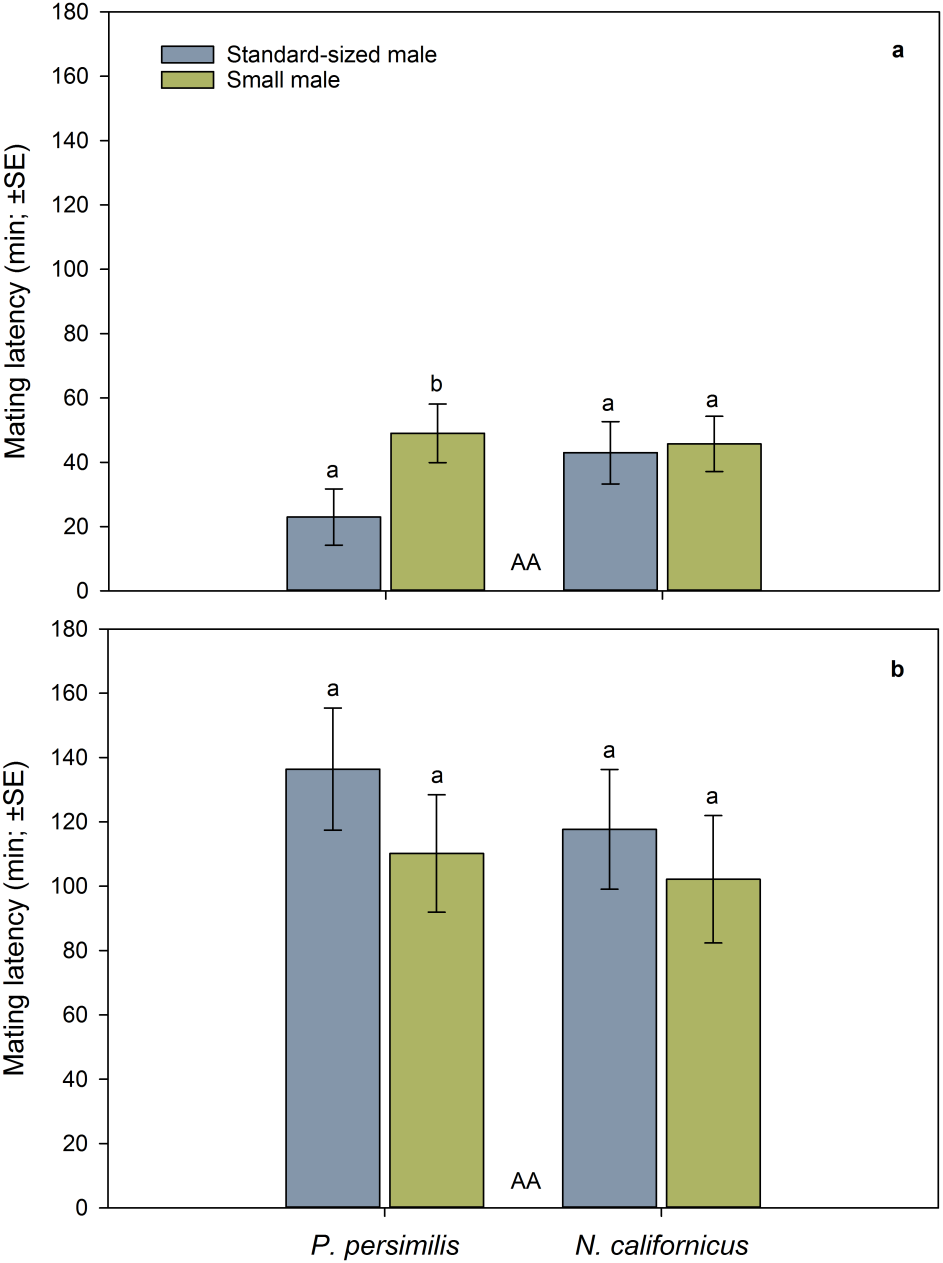
Influence of male body size and mating sequence on mating latency. First (a) and second (b) mating latency of doubly mating *P*. *persimilis* (*n* = 52) and *N*. *californicus* (*n* = 49) females, in dependence of the mating sequence of small and standard-sized males. Same capital letters between bars indicate non-significant differences between species across mating sequences (GLM). Different lower case letters on top of bars indicate significant differences between small and standard-sized male mates within species (LSD following GLM; *P* < 0.05).

**Fig 4:**
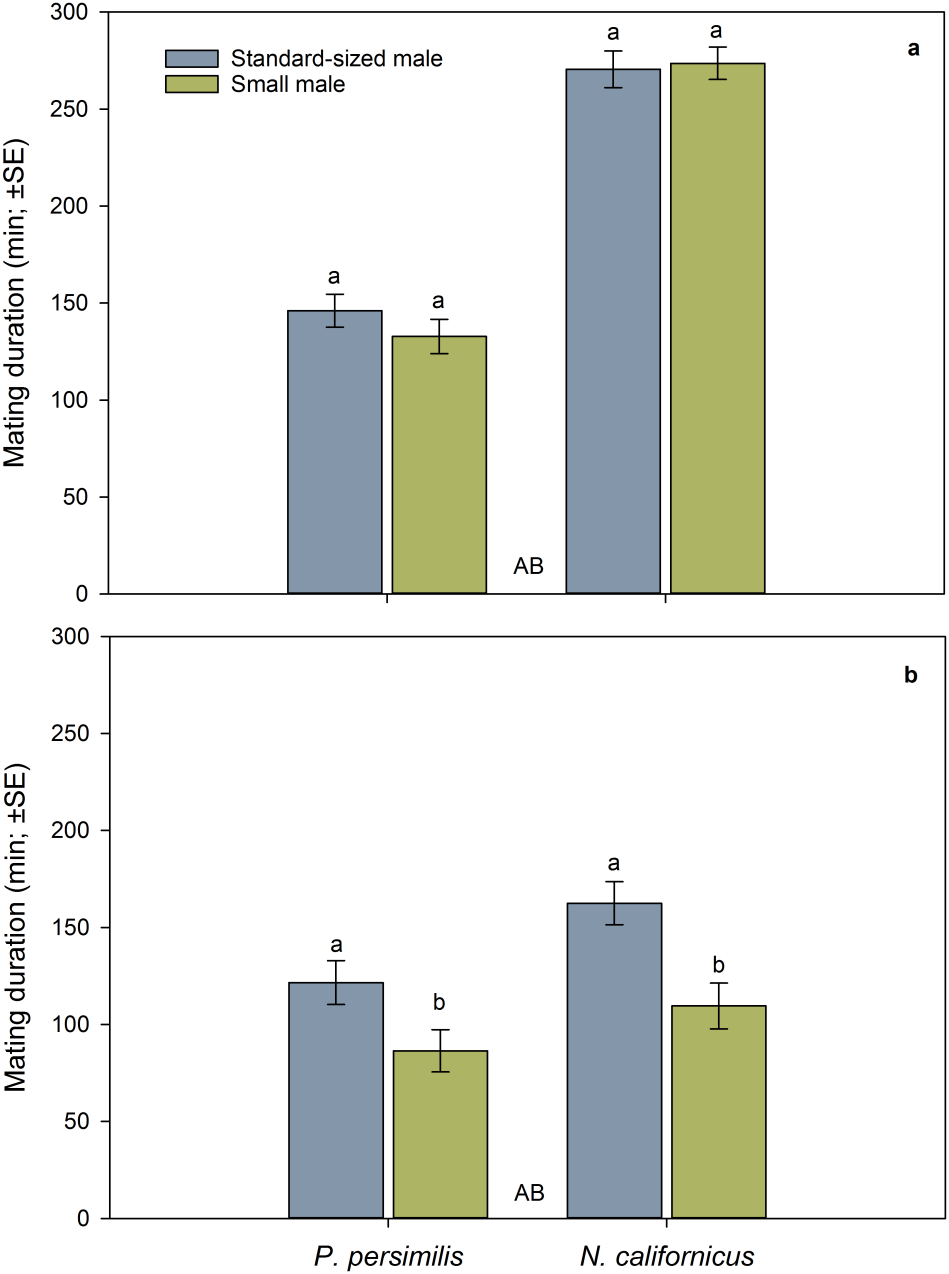
Influence of male body size and mating sequence on mating duration. First (a) and second (b) mating duration of doubly mating *P*. *persimilis* (*n* = 52) and *N*. *californicus* (*n* = 49) females, in dependence of the mating sequence of small and standard-sized males. Different capital letters between bars refer to differences between species across mating sequences (GLM; *P* < 0.01). Different lower case letters on top of bars indicate significant differences between small and standard-sized male mates within species (LSD following GLM; *P* < 0.05).

The first mating date (Fig 2a) was neither influenced by species nor male mate body size nor the interaction between species and male size (Table 1). The latency to first mating (Fig 3a) was similar in both species but affected by male mate body size and its interaction with species (Table 1). The latter indicates that small males were later accepted as mates than standard-sized males in *P*. *persimilis* but not in *N*. *californicus* (Fig 3a). The duration of the first mating was longer in *N*. *californicus* than *P*. *persimilis*, independent of male mate body size and the interaction between species and male size (Table 1, Fig 4a).

**Table 1:** Results of generalized linear models (GLM) testing the influence of species and mating sequence on mating behavior, egg production, and paternity success in doubly mating females of the predatory mites *Phytoseiulus persimilis* and *Neoseiulus californicus*. Single virgin females were either offered first a standard-sized and second a small male mate or offered first a small and second a standard-sized male mate. Significant P-values are highlighted in bold.

Variable	Species^1^		Mating sequence^2^		Species*mating sequence	
	*Wald ӽ*_*1*_^*2*^	*P*-value	*Wald ӽ*_*1*_^*2*^	*P*-value	*Wald ӽ*_*1*_^*2*^	*P*-value
1^st^ mating date	0.007	0.94	2.536	0.11	1.657	0.20
1^st^ mating latency	3.063	0.08	6.646	**0.01**	4.781	**0.03**
1^st^ mating duration	227.705	**<0.001**	0.337	0.56	0.874	0.35
2^nd^ mating date	9.360	**<0.001**	0.013	0.91	8.177	**<0.001**
2^nd^ mating latency	0.486	0.49	1.230	0.27	0.051	0.82
2^nd^ mating duration	8.049	**0.005**	15.244	**<0.001**	0.624	0.43
Total eggs	52.977	**<0.001**	0.264	0.61	0.044	0.83
Eggs produced after re-mating	33.834	**<0.001**	0.001	0.97	3.888	**0.05**
1^st^ mate’s paternity share after re-mating	14.823	**<0.001**	9.317	**0.002**	9.980	**0.002**

^1^*Phytoseiulus persimilis* vs. *Neoseiulus californicus*

^2^1^st^ standard-sized male and 2^nd^ small male vs. 1^st^ small male and 2^nd^ standard-sized male

The second mating date (Fig 2b) was influenced by species and its interaction with male mate body size but not by male mate body size as main effect (Table 1). *P*. *persimilis* mated earlier with small than standard-sized males, whereas *N*. *californicus* mated earlier with standard-sized than small males. The latency to second mating (Fig 3b) was neither influenced by species nor male mate body size nor the interaction between species and male size (Table 1). The duration of the second mating (Fig 4b) was longer in *N*. *californicus* than *P*. *persimilis* and longer with standard-sized than small males in both species (Table 1).

**Fig 5:**
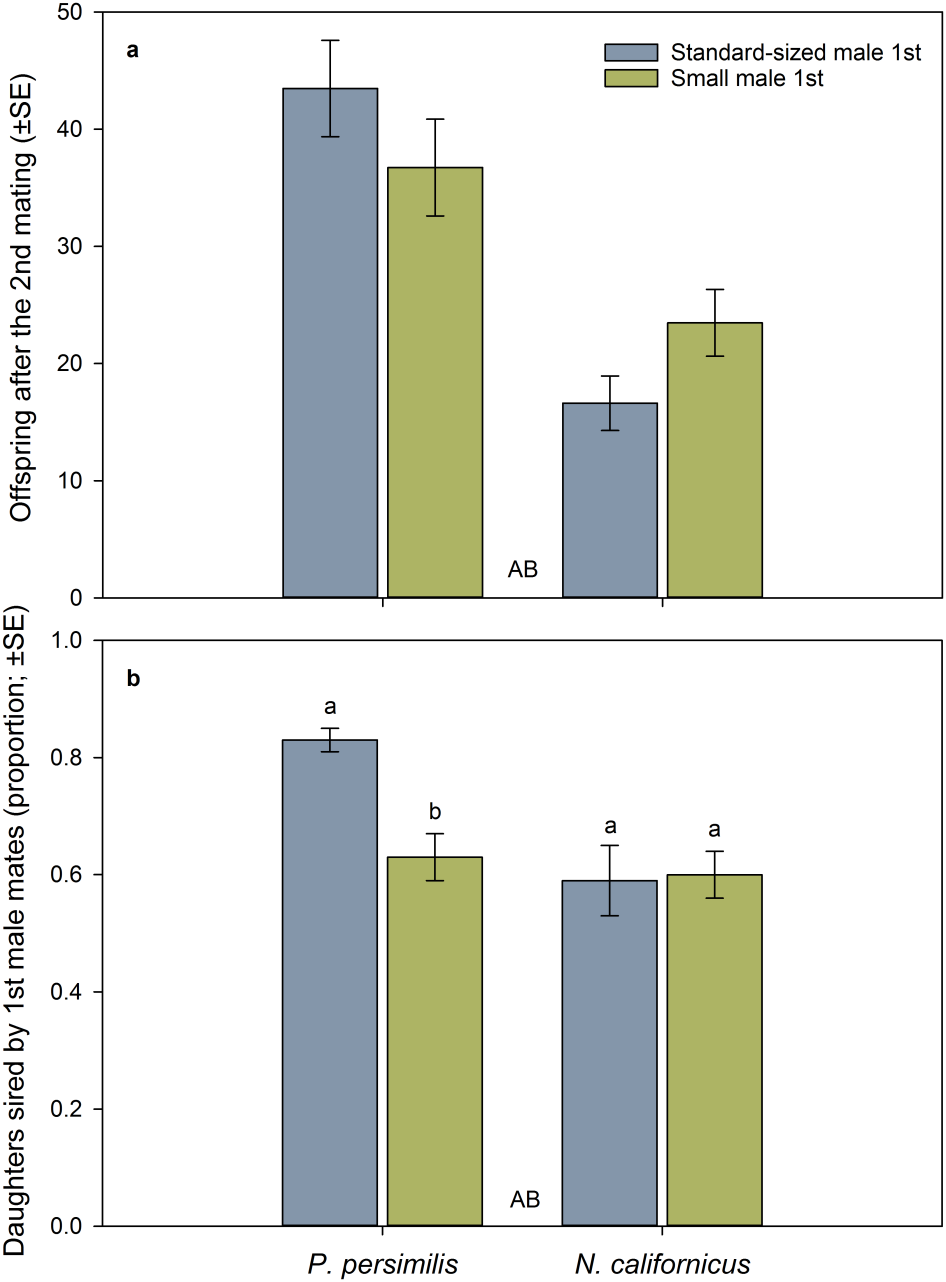
Influence of male body size and mating sequence on post re-mating offspring production and paternity. Number of offspring (a) and proportion of daughters sired by first male mates (b) after re-mating by doubly mated females of *P*. *persimilis* [*n* = 50 for (a) and *n* = 22 for (b)] and *N*. *californicus* [*n* = 44 for (a) and *n* = 17 for (b)], in dependence of the mating sequence of small and standard-sized males. Different capital letters between bars refer to significant differences between species across mating sequences (GLM; *P* < 0.001). In (a), GLM revealed *P* = 0.05 for species*mating sequence. In (b), different lower case letters on top of bars indicate significant differences between small and standard-sized male mates within species (Sidak following GLM; *P* < 0.05).

*Phytoseiulus persimilis* females produced more eggs in total (mean ± SE; standard-sized male as 1^st^ mate 63.72 ± 3.20 vs. small male as 1^st^ mate 61.69 ± 3.33) than *N*. *californicus* females (42.69 ± 3.39 vs. 41.84 ± 1.55), independent of the mating sequence of small and standard-sized males (Table 1). After re-mating, *P*. *persimilis* females produced more offspring than *N*. *californicus* females, independent of the mating sequence as a main effect (Table 1). The significant species by mating sequence interaction indicates that *P*. *persimilis* produced more eggs after the second mating when the second mate was small, whereas the reverse was the case in *N*. *californicus* (Table 1, Fig 5a). The proportion of daughters sired by first mates after female re-mating was higher in *P*. *persimilis* than *N*. *californicus* and higher for first standard-sized than first small male mates. However, the latter was only true for *P*. *persimilis* but not *N*. *californicus*, as indicated by the significant interaction between species and mating sequence (Table 1, Fig 5b).

## Discussion

Our study suggests that the level of polyandry is an important co-determinant of the level of male body size plasticity in the predatory mites *P*. *persimilis* and *N*. *californicus*. Male body size plasticity seems constrained by the costs of deviations from standard body size in mating interactions. In the less polyandrous *P*. *persimilis*, deviations from standard body size are more costly for males, because (i) females have non-random mate preferences and prefer standard-sized to small males when given a choice [34], (ii) females mate earlier with standard-sized than small males under no-choice (present study), (iii) small males transfer fewer spermatophores than standard-sized males (present study), (iv) small males fertilize fewer eggs per mate and have a lower lifetime reproductive success than standard-sized males [36], and (v) first standard-sized males sire a higher proportion of offspring in doubly mating females than do first small males (present study). These costs of being small do not apply to males in the moderately polyandrous *N*. *californicus* ([34,36], present study).

Female re-mating opens the chance of sperm competition and cryptic female choice [46] but is disadvantageous from the first males’ perspective, because potentially reducing their proportion/number of sired offspring and allowing replacing their sperm. Females of both *P*. *persimilis* and *N*. *californicus* re-mated more likely when the first mate was small, opening higher chances for second mates to sire offspring, than when the first male was standard-sized. However, in *N*. *californicus* the risk of first small, relative to first standard-sized, mates to lose paternity to later mates is relaxed, as compared to *P*. *persimilis*, because the females mate more often, providing more chances to gain paternity, and later mates have a higher share in siring offspring [38]. In *N*. *californicus*, first standard-sized and small mates sired similar proportions of offspring after female re-mating. In contrast, in *P*. *persimilis*, first standard-sized mates sired a higher proportion of offspring after female re-mating than first small mates, buffering the risk of losing paternity. Similar to our observations, females of the seed beetle *Stator limbatus* and the bean beetle *Callosobruchus maculatus* re-mate later, or have a lower re-mating propensity, when first mating with a large male than when first mating with a small male [47,48].

In *P*. *persimilis*, first standard-sized males sired a higher proportion of daughters than first small males after female re-mating, which ultimately indicates higher success of first standard-sized than small males in post re-mating sexual selection. Proximately, the advantage of first standard-sized over first small male mates was likely mediated by (i) first standard-sized males transferring more spermatophores than first small males, and (ii) females mating first with a standard-sized and second with a small male producing more eggs after re-mating than females first mating with a small and second with a standard-sized male. Regarding the latter, the opposite was the case in *N*. *californicus*, which was likely due to different timing of re-mating. In *P*. *persimilis* re-mating occurred around two days earlier, but in *N*. *californicus* three days later, with second small than second standard-sized males. Assuming that sperm competitiveness depends, among others, on sperm age [49], earlier re-mating with second small than standard-sized males in *P*. *persimilis* provided an advantage for first standard-sized over first small males in sperm competition/cryptic female choice after re-mating.

Possible, mutually non-exclusive, explanations why in *P*. *persimilis* standard-sized males were more likely, but later, accepted as re-mates than were small males are: (i) standard-sized males are choosier in who they approach for mating, because, in choice situations, they are superior in male-male competition and preferred by the females to small males [34]; (ii) re-mating females delay the second mating with standard-sized males when they first had a small male, to optimize indirect benefits, such as increasing the genetic variability of their offspring [2]; small males produce fewer offspring and their paternity contribution might become very low if the females mate too early with second standard-sized males; (iii) females accepting small males as second mates are indiscriminate and therefore accept second mates soon after the first mating; choosy females take time to decide whether re-mating pays or not and thus accept second mates later.

One decisive ultimate reason why *P*. *persimilis* females re-mated more likely with standard-sized following small than small following standard-sized males is the lower egg fertilization potential of small than standard-sized males [36]. Females mating first with a small and second with a standard-sized male produced as many eggs as females mating first with a standard-sized and second with a small male. Thus, re-mating provided a direct material benefit to females that first mated with a small male, allowing to produce more eggs than if staying singly mated. In contrast, females mating first with a standard-sized and second with a small or standard-sized male produced as many eggs as females once mating with a standard-sized male [36,38]. Apparently, the latter type of re-mating females solely target indirect benefits. Small males commonly transferred only one spermatophore, whereas standard-sized males mostly transferred two spermatophores, allowing filling both spermathecae of the females. Considering similar ejaculate mass of small and standard-sized males, filling only one or both spermathecae is under female control but not a matter of sperm limitation of small males. Obviously, *P*. *persimilis* females mating first with a small male left one spermatheca empty to leave room for filling by a second male.

Overall, our findings support the hypothesis that the lower male body size plasticity of *P*. *persimilis* than *N*. *californicus* is sexually selected. There is no indication that the interspecific difference in male body size plasticity, coupled with similarity in female body size plasticity, is naturally selected. On the contrary, due to the higher specialization of *P*. *persimilis* in exploiting the shared spider mite prey, one would expect higher body size plasticity in *P*. *persimilis* than *N*. *californicus*. Spider mites are patchily distributed on their host plants and constitute a highly ephemeral prey [50]. Environmental heterogeneity and fluctuations are major determinants favoring higher body size plasticity [26,27,29]. At large, food availability is more homogenous and less fluctuating for the generalist *N*. *californicus* than the spider mite specialist *P*. *persimilis* because *N*. *californicus* can use various food sources and thus experiences food limitation less likely and less frequently than does *P*. *persimilis*. Apart from differences in diet specialization, these two co-occurring species have similar ecological niche requirements, providing no indication for naturally selected differences in male body size plasticity.

## Conclusions

Based on the results of our study and those of pertinent previous studies, which showed that standard-sized males fertilize more eggs than small males and are preferred in female choice in *P*. *persimilis* but not *N*. *californicus*, and the absence of any clue pointing at a role of natural selection, we suggest that the interspecific difference in male body size plasticity, at interspecific similarity of female body size plasticity, is sexually selected. Our study provides a pioneering indication of the influence of sexual selection on plasticity of male phenotypes, suggesting that low level of polyandry may constrain the level of phenotypic plasticity of male body size.

## Supporting information

S1 Table Raw data of the experiments and paternity analysis. (XLSX)
